# Intraosseous concentration and inhibitory effect of different intravenous cefazolin doses used in preoperative prophylaxis of total knee arthroplasty

**DOI:** 10.1007/s10195-015-0370-y

**Published:** 2015-08-02

**Authors:** Chayanin Angthong, Pongpaibool Krajubngern, Warawut Tiyapongpattana, Boonchana Pongcharoen, Piya Pinsornsak, Nattapol Tammachote, Wanna Kittisupaluck

**Affiliations:** Department of Orthopaedics, Faculty of Medicine, Thammasat University, Pathum Thani, 12120 Thailand; Department of Chemistry, Faculty of Science and Technology, Thammasat University, Pathum Thani, Thailand; The Surgical Unit, Thammasat University Hospital, Pathum Thani, Thailand

**Keywords:** Cefazolin, Knee arthroplasty, Prophylaxis, Intraosseous concentration, Inhibitory, Effect

## Abstract

**Background:**

The aim of this study was to compare the intraosseous concentrations and the inhibitory effects on the growth of *Staphylococcus aureus* of 1 g versus 2 g of intravenous (IV) prophylactic cefazolin in total knee arthroplasty (TKA).

**Materials and methods:**

Eighteen patients (21 knees) with primary knee osteoarthritis were divided into two groups receiving 1 g (12 patients: 14 knees) versus 2 g (six patients: seven knees) IV prophylactic cefazolin prior to the incision in TKA. Subchondral bone samples (proximal tibia, distal femur) were taken during the operation. These samples were analyzed for intraosseous concentration of cefazolin and their inhibitory effects on the growth of *S. aureus*, using high-performance liquid chromatography (HPLC) and agar disc diffusion bioassays.

**Results:**

The mean intraosseous concentration in the 2 g dose group was significantly higher than in the 1 g dose group in the proximal tibia (*p* = 0.007) and distal femur (*p* = 0.016). There were no significant differences between the two groups in terms of mean inhibitory effects in the proximal tibia or distal femur (*p* > 0.05). No significant correlations were found between the intraosseous concentrations and inhibitory effects in the proximal tibia (*r* = 0.18, *p* = 0.52) and distal femur (*r* = −0.29, *p* = 0.30).

**Conclusion:**

IV cefazolin at a dose of 2 g produced greater intraosseous concentrations overall than a dose of 1 g. However, the higher intraosseous concentrations did not correlate with higher inhibitory effects.

**Level of evidence:**

Level III.

## Introduction

Prophylactic antibiotics are known to reduce the risk of perioperative and/or postoperative infection [1–3]. However, some previous studies had reported that systemic antibiotics may not prevent all postoperative infections [4–6]. Moreover, conventional systemic dosages may not provide adequate tissue concentrations against more resistant organisms, such as coagulase-negative staphylococci [7]. The current literature recommends the intravenous (IV) administration of cefazolin, 1–2 g [1], within 1 h prior to making the incision. This antibiotic may be repeated every 2–5 h during the operation and should be stopped within 24 h following the operation [8, 9]. However, little is known about the differences in the intraosseous concentrations of cefazolin or the inhibitory effects on the growth of *Staphylococcus aureus* between an IV dose of 1 g and a dose of 2 g.

This study aims to compare the intraosseous concentrations and the inhibitory effects on the growth of *S. aureus* of IV prophylactic cefazolin at dosages of 1 versus 2 g in total knee arthroplasty. At this point, we hypothesize that the intraosseous concentrations and the inhibitory effects in the group given cefazolin at a dose of 2 g are possibly higher than those in the group given a dose of 1 g.

## Materials and methods

During the period between May, 2011 and February, 2013, patients with primary knee osteoarthritis were recruited to participate in this study. The inclusion criteria were patients with primary knee osteoarthritis while the exclusion criteria included patients with post-traumatic or post-infectious knee conditions, allergies to cephalosporin or penicillin, serum creatinine levels >1.5 mg%, creatinine clearance <55 ml/min, probenecid intake, post-steroid treatment, chemotherapy, those who were post-knee arthroplasty or high tibial osteotomy, immunocompromised hosts, and those with deleterious medical conditions. Eighteen patients were recruited into our study using the inclusion and exclusion criteria. The 18 patients (21 knees) were divided into two groups in accordance with their IV prophylactic cefazolin dosages (cefazolin M. H., M & H Manufacturing Co., Ltd., Thailand) of 1 g (12 patients: 14 knees, which were operated by B.P. and P.P.) versus 2 g (six patients: seven knees, which were operated by N.T.) administered before the incision was made in their total knee arthroplasty. All patients’ baseline data were prospectively collected, including the intraosseous concentrations of cefazolin and the inhibitory effects on the growth of *S. aureus,* which were prospectively collected from further analyses. The group with the IV cefazolin dose of 1 g consisted of nine females and three males with a mean age of 70.1 ± 4.6 years (61–79 years of age). During the same period we retained a group with an IV cefazolin dose of 2 g. This group consisted of five females and one male with a mean age of 68.4 ± 3.0 years (64–73 years of age). There were no significant differences in the mean age and genders between the two groups (*p* = 0.40 for age, *p* = 1.00 for gender). The mean weights of the patients were 61.2 ± 8.4 kg for the group with a 1 g dose and 62.0 ± 8.2 kg for the group with a dose of 2 g (*p* = 0.84).

All patients were prepared for the TKA operation with standard, sterile technique. They were administered IV cefazolin, at a dose of 1 or 2 g, prior to tourniquet inflation and before the incision had been made. After performing knee arthrotomy and bone cutting at the distal femur and at the proximal tibia, our experienced knee surgeons, B.P., P.P., and N.T. (with the same level of experience), provided bone from the segments that had been removed from each patient to a researcher (P.K.) who extracted only subchondral bone at a size of 2.5 × 2.5 mm for further analyses while under sterile technique. The period of time between IV cefazolin injection and sample collection was recorded.

Each sample was processed by the antibiotic broth elution assay [10]. The solution from a previous process was further analyzed for concentration using high-performance liquid chromatography–photodiode array detection [HPLC–DAD; Shimadzu (Nexara LC-30A)]. The unit of this intraosseous concentration was ‘µg/g’, which was derived from the comparison of a 1 g sample of the subchondral bone. A validation study was done of the extraction and HPLC–DAD technique using bone samples analyzed with known concentrations of cefazolin. All samples were analyzed by a specialist (W.T.) who was blinded to the IV cefazolin dose in each sample.

The bioactivity of each sample was determined using an agar disc diffusion bioassay with *S. aureus* (ATCC 25923) [10]. The antibiotic bioassay was to determine the inhibitory effect of intraosseous cefazolin, which was compared with the standard minimal inhibitory concentration (MIC) of serum cefazolin (30 µg/ml). The technique was based on the inhibitory activity of discs (Oxoid, UK) containing a standard concentration of cefazolin. Standard paper discs and samples were placed on *S.aureus*-seeded agar (Muller Hinton Agar, BD, USA) and incubated for 18 h at 37 °C. All samples were analyzed by our researcher (N.M.) who was blinded to the IV cefazolin dose in each sample. The standard MIC of serum cefazolin would inhibit the growth of *S. aureus* at least 18 mm from the center of the sample. All samples were analyzed by a staff person in the laboratory who was also blinded to the IV cefazolin dose used in each sample.

### Statistical analyses

Statistical analysis was performed using the SPSS software version 13.0 (SPSS Inc., Chicago, IL, USA). ANOVA was used to analyze the statistical significance of the differences in the values of the intraosseous concentration of cefazolin (µg/g) and the inhibitory effect on *S. aureus* growth on the agar disc (mm) between the two different groups of IV cefazolin doses (1 versus 2 g). The correlations between the intraosseous concentrations of cefazolin (µg/g) and the inhibitory effects of *S. aureus* growth on agar disc (mm) were analyzed and interpreted via Pearson’s correlation coefficient (*r*). Categorical variables were analyzed using the Chi-squared test. The level of significance was set at *p* < 0.05.

## Results

The mean times between cefazolin injection and sample collection were 50.1 ± 6.5 min for femur and 53.1 ± 6.5 min for tibia in the group with IV cefazolin 1 g, compared with 58.9 ± 23.1 min for femur and 67.9 ± 23.1 min for tibia in the group with IV cefazolin 2 g. There were no significant differences in these mean times for the same corresponding areas of sample collection between the two groups (femur, *p* = 0.32; tibia, *p* = 0.11).

Total mean intraosseous concentrations of cefazolin were 26.6 ± 11.7 µg/g in the proximal tibia and 30.5 ± 19.5 µg/g for the distal femur. The mean intraosseous concentrations of the group receiving cefazolin 2 g were significantly higher than the group receiving cefazolin 1 g in the proximal tibia (*p* = 0.007) and distal femur (*p* = 0.016) (Table 1).

**Table 1:** Intraosseous concentrations of cefazolin in the two groups

Parameters	IV cefazolin 1 g	IV cefazolin 2 g	*P* value
Cefazolin concentration at proximal tibia (µg/g)	21.4 ± 11.4	35.5 ± 5.1	0.007*
Cefazolin concentration at distal femur (µg/g)	22.6 ± 8.7	44.1 ± 25.8	0.016*

* Significant difference

From Pearson’s correlation analyses, there were no significant correlations between the levels of intraosseous concentration and inhibitory effects seen in the proximal tibia (*r* = 0.18*, p* = 0.52) and distal femur (*r* = −0.29, *p* = 0.30).

The total mean inhibitory effects of cefazolin were 10.0 ± 5.4 mm for the proximal tibia and 11.6 ± 5.4 mm for the distal femur. The mean inhibitory effects in the two groups (cefazolin 1 g versus 2 g at both proximal tibia and distal femur) were less than 34.6 ± 0.5 mm, which was shown to be the mean inhibitory effect of the standard MIC of serum cefazolin (30 µg/ml) in this present study (Table 2). There were no significant differences between the two groups in terms of the mean inhibitory effects at the proximal tibia or distal femur via the analyses on agar disc diffusion bioassay (proximal tibia, *p* = 0.33; distal femur, *p* = 0.63).

**Table 2:** Mean inhibitory effects in the two groups

Parameters	IV cefazolin 1 g	IV cefazolin 2 g	*P* value
Mean inhibitory effect at proximal tibia (mm)	11.1 ± 6.1	8.4 ± 4.2	0.33
Mean inhibitory effect at distal femur (mm)	12.2 ± 6.1	10.9 ± 4.6	0.63

## Discussion

Although current literature recommends administration of a dosage of intravenous (IV) cefazolin of 1–2 g [1] as the dose for prophylaxis in TKA, there is, as far as we know, a lack of basic research that has shown the actual intraosseous concentrations and inhibitory effects of cefazolin at those recommended doses. Our study is possibly one of the earliest reports on the importance of these aspects. Based on the present study, we found that the mean intraosseous concentrations in the group receiving 2 g of cefazolin were significantly higher than in the group receiving 1 g, at the proximal tibia (*p* = 0.007) and distal femur (*p* = 0.016). However, the mean intraosseous concentrations of the 1 g group (22.6 µg/g) and the 2 g group (44.1 µg/g) at the distal femurs were higher than the concentrations seen in a previous study (9.2 µg/g) [7].

On the other hand, there were no significant differences between the two groups in terms of the mean inhibitory effects at either the proximal tibia or distal femur via agar disc diffusion bioassay (proximal tibia, *p* = 0.33; distal femur, *p* = 0.63). Although a previous report had shown that high-dose cefazolin could be used for prophylaxis in an animal study [11], surgeons should keep in mind that, based on our findings, when they consider IV cefazolin at a dose of 2 g for preoperative prophylaxis, it should be used with care. This is especially true for patients with reduced renal function. In addition to the results that showed no significant differences between the two groups in terms of mean inhibitory effects, we found that the mean inhibitory effects in the two groups (cefazolin dose of 1 versus 2 g at both the proximal tibia and distal femur) were less than the mean inhibitory effect of the standard MIC of serum cefazolin at 30 µg/ml. Pearson’s correlation analyses showed that there were no significant correlations between the intraosseous concentrations and the inhibitory effects in either the proximal tibia (*r* = 0.18*, p* = 0.52) or distal femur (*r* = −0.29, *p* = 0.30). Higher cefazolin dosages would not provide higher inhibitory effects. At this point, we hypothesize that the standard MIC of intraosseous cefazolin may not be directly related to the inhibitory effects, which are different from the relationship between the standard MIC of serum cefazolin and its inhibitory effects via the agar disc diffusion bioassay. In addition, the intraosseous area may contain a threshold inhibitory effect of cefazolin that gives no greater inhibitory effect from a higher dose of cefazolin. Further studies are needed to test the inhibitory effects of intraosseous cefazolin directly with the standard MIC of intraosseous cefazolin rather than relative tests with the standard MIC of serum cefazolin.

There were some limitations in the present study. First of all, bone resection at the distal femur and at the proximal tibia for the analyses of intraosseous concentrations and inhibitory effects of cefazolin were performed only once after knee arthrotomy. There was no subsequent bone sampling for further analyses of the same parameters at the end of the operation. Therefore, the change in intraosseous concentration of cefazolin over the operating time could not be determined at this point. A possible decrease in concentration at the end of wound closure, including the periods when suturing and insertion of drains may be a source of contamination, was not clarified in the study. The protective effect of the higher dose (2 g) of cefazolin may be more beneficial than that of the lower dose (1 g) due to the longer persistence of the inhibitory effect, particularly in the final period of the operation. A previous study found that biofilm formation could develop for up to 1–2 days [12]; therefore, hypothetically, the higher dose (2 g) of cefazolin might be more beneficial than the lower dose of 1 g. Second, the recommendations for surgeons from the results of the present study might appear to be weak because of the lack of evidence concerning relationships between the intraosseous concentration of cefazolin, its inhibitory effect and the related infection. This issue relates to the previous limitation mentioned, as there was no clarification of those relationships during the final period of the operation. Future study may help to clarify these issues.

IV cefazolin at a dose of 2 g provides greater intraosseous concentrations in both the proximal tibia and distal femur than does a dose of 1 g. However, its higher intraosseous concentration does not correlate with higher inhibitory effects. Surgeons have to weigh the advantages and disadvantages when considering IV cefazolin at a dose of 2 g for preoperative prophylaxis in total knee arthroplasty.
